# Person‐Directed Staffing and Quality of Life: Links Between Staff Choice, Perceived Staff Stability and Community Participation for Individuals With IDD in the United States

**DOI:** 10.1111/jar.70265

**Published:** 2026-06-25

**Authors:** James Houseworth, Renáta Tichá, Roger J. Stancliffe, Sandra L. Pettingell, Yi‐Chen Wu

**Affiliations:** ^1^ Institute on Community Integration University of Minnesota Minneapolis Minnesota USA

**Keywords:** choice, community inclusion, intellectual disability, National Core Indicators, staff stability

## Abstract

**Background and Aims:**

Direct support professionals (DSPs) are essential to individuals with intellectual and developmental disabilities (IDD), yet staff turnover remains a significant concern. This study examined whether individuals with IDD in the United States are involved in choosing their staff, how they perceive staff turnover and whether perceived staff stability is associated with quality of life.

**Methods:**

Data from the 2022–2023 National Core Indicators–In Person Survey were analysed to assess relationships between staff choice, perceived staff stability and outcomes related to everyday choice, support‐related choice and alignment of community activities with personal preferences.

**Results:**

Individuals involved in choosing their staff were more likely to report greater staff stability. Perceived staff stability was associated with better alignment between community participation and personal preferences but was not significantly associated with choice‐making outcomes.

**Conclusion:**

Findings support person‐directed service models and highlight the value of involving individuals with IDD in staffing decisions to promote staff stability and person‐centred community participation.

## Lay Summary

- People with intellectual and developmental disabilities who helped choose their support staff were more likely to feel that their staff stayed longer.
- When staff were more stable, people were more likely to participate in community activities that matched their personal interests.
- The findings highlight the importance of involving people with disabilities in decisions about who supports them, which may improve aspects of their quality of life.

## 1 Introduction

Direct support professionals (DSPs) are essential to people with intellectual and developmental disabilities (IDD) because of the assistance they provide with daily activities and community participation. DSPs have a range of complicated job duties, including assisting with relationships, networking, communication, personal care, health and safety, transportation, advocacy, managing finances, community living, crisis prevention, household tasks, education on self‐care skills and promoting self‐determination (Bogenschutz et al. 2014; Hasan 2013; Hewitt and Lakin 2001; Hewitt and Larson 2007; Hewitt et al. 2008; Robbins et al. 2013; Wright 2009). All these job tasks are necessary to expand and maintain quality of life among people with IDD. Further, DSPs require deep knowledge of an individual's needs and preferences to provide person‐centred support. This level of understanding is achieved over time and requires the continuity of interactions that is disrupted by high staff turnover.

### 1.1 Staff Turnover

The cause and extent of DSP turnover in IDD services have been widely studied. Direct support is among the most prevalent jobs in the country, with nearly 5 million DSP positions in the United States (Hall et al. 2024; Campbell et al. 2021) and an additional estimated 2.3 million new DSPs required by 2028 (Campbell et al. 2021). Issues such as low wages, a lack of benefits and full‐time hours, inadequate supervision, burnout and a lack of social support contribute to high turnover rates (Houseworth et al. 2020; Pettingell et al. 2023; Hall et al. 2024; Ejaz et al. 2015). National Core Indicators (NCI) State of the Workforce 2023 data indicated an average annual turnover ratio of 39.7% (range 23.6% to 48.6%) for US provider agencies serving individuals with IDD who participated in the survey. Vacancy rates averaged 12.2% (range 6.9% to 18.2%) for full‐time DSPs and 14.6% (range 4.6% to 22%) for part‐time DSPs (National Core Indicators Intellectual and Developmental Disabilities [NCI‐IDD] 2024). A nongovernmental organization responsible for assisting employers recruit the adult long‐term care workforce in England estimated that in 2016 annual turnover was at 26% in care homes and 33% in home care agencies, compared with 15% in the English workforce (Murphy 2016).

### 1.2 Consequences of DSP Turnover

High staff turnover is a financial burden to organizations, compromises the quality of services and negatively impacts the wellbeing of other staff (Keesler and Troxel 2020). While turnover is a problem for provider agencies in terms of recruitment and training new staff and the extra duties and stress it places on remaining staff, there is much less evidence on the effect on people with IDD and the outcomes they experience. Such effects are presumably due to issues with new staff: (a) being unfamiliar with the individual and their support needs and preferred outcomes, and (b) not (yet) having developed a personal relationship with each person served as a trusted support provider. Further, new staff may have little to no experience in the IDD field and therefore have fewer skills and less knowledge on how to support people with IDD. In settings like group homes where DSPs may work alone, there may be no experienced co‐workers on hand to guide or train new staff. Thus, a higher turnover rate likely will have a negative impact on the quality of support for people with IDD, especially for people with complex needs.

DSP turnover is of concern due to the critical role DSPs play in supporting and broadening opportunities for community living, enhancing the quality of life for individuals with IDD (Robbins et al. 2013). Friedman (2018) determined whether the person receiving services had experienced changes in DSP staff over the past 2 years and examined the association with their quality‐of‐life outcomes. The analyses demonstrated a significant relationship between staff turnover and important individual quality of life outcomes (e.g., safety, health, choice‐making, community participation). In all cases, turnover had a negative impact on the outcomes assessed.

Friedman's (2018) data came from the *Personal Outcome Measures* (The Council on Quality and Leadership 2017). This measure involves separately interviewing the person receiving services and a proxy (e.g., family, friend) after which the interviewer uses this information to determine if each outcome is present. That is, the findings are based on a mix of self‐report and proxy responses, and in relation to staff turnover ‘the certified interviewer decides if the person has experienced changes in DSP staff in the past 2 years’ (Friedman 2018, 241), an objective measure of staff turnover. By contrast, the National Core Indicators In‐Person Survey (NCI‐IPS) item used in the current study simply asks the person whether they think that their staff change too often.

### 1.3 Measurement of Staff Turnover

Much of the research on turnover in the United States relies on agency‐level administrative data (i.e., separations reported by the agency of DSPs leaving their employment) and subsequent impact separations have on the agency's finances and burnout of staff working in understaffed settings (e.g., Pettingell et al. 2022). Further, these measures do not identify the impact of turnover on the individual, which can vary depending on their personal perceptions, needs, goals, relationship with that DSP and other factors (e.g., living arrangements). Apart from Friedman (2018), this level of individual person‐centred staff turnover has rarely been examined by researchers. The impact of turnover as perceived by the people with IDD directly affected by it is essential information that gets to the heart of the problem. The NCI‐IPS staff turnover variable used in the current study is person centred and subjective in that it measures each individual with IDD's view on whether their staff change too much by directly asking them (see the Section 3 for more details). This approach captures staff turnover from the perspective of the individual service user and reflects *both* DSPs that leave the agency (the usual measure of turnover at the agency level) *and* the staff turnover experienced by the individual when a DSP transfers to another part of the same agency (an aspect not captured by agency‐level turnover measures).

### 1.4 Choice of Staff and Staff Turnover

Choosing your own staff is an option only available to some service users (e.g., those who use self‐directed services) and is frequently called *employer authority*. When fully developed, employer authority involves the freedom to hire, train, supervise, schedule and in some cases to dismiss one's support professionals, usually within state guidelines (Friedman 2025). In some cases, people can choose to hire people they already know and trust such as family members or friends as DSPs. Choosing your own staff could well lead to greater compatibility between staff and the person with IDD, which in turn may result in lower staff turnover. For example, Murray et al. (2022, 13) in the UK found that DSPs reported that the most important factor regarding job retention was ‘the relationship of the staff member with the person they supported’. Further, it seems possible that choosing staff and greater compatibility/better relationships may both lead to better individual outcomes. To our knowledge, these issues have not been examined previously in quantitative IDD research.

One related topic to choosing staff in IDD services in the United States and in other national contexts is self‐direction of services. Self‐directed services are a long‐term care model that allow individuals with disabilities to manage their own care, rather than relying on case management (Medicaid.gov). When we discuss individuals choosing their own staff (or having a role in it), we have included all participants who choose their staff regardless of self‐directed services status. We did not include a self‐direction variable in the current study, but we explore the relationship between self‐direction, choice of staff and perceived staff turnover in a companion paper (Stancliffe et al. 2026).

In this paper we wanted to fill this gap in the literature by exploring whether an individual's involvement in choosing their staff is related to staff turnover. In previous research using NCI‐IPS, choice of staff has largely been combined with other choices as part of a larger concept of *support‐related choice* (Houseworth et al. 2018; Tichá et al. 2012), but we chose to analyse choice of staff separately and exclude this item from the support‐related choice scale. There are studies that report on the benefits of self‐determination and autonomy of adults with IDD with respect to making their own choices and decisions over the things that matter to them (Wehmeyer and Abery 2013; Carey et al. 2023). However, very few studies address choice of staff by service users with IDD specifically and of those, none have examined its relationship to staff turnover. In the Spanish context, Pallisera et al. (2021) reported that professional practices of a service agency, particularly the level of autonomy organizations give to people with IDD, determined how these individuals perceive their ability to choose staff. Swaine et al. (2016) in a qualitative study focused on self‐directed services found that many participants with intellectual and physical disabilities appreciated the ability to manage their care and hire their staff, including employing a relative or friend who they were familiar with. Despite reporting some problems with staff (e.g., difficulty supervising staff; feeling uncomfortable dismissing staff dependent on the earnings), most participants reported many positive experiences with their staff.

### 1.5 Choice and Community Participation

Community participation and choice are both likely to be impacted by staff turnover, as reported by Friedman (2018). Previous research utilizing NCI‐IPS data (Houseworth et al. 2018; Tichá et al. 2012), as the current study does, found two types of choice: *everyday choice* and *support‐related choice*. Similarly, Bush and Tassé (2017) identified two scales: long‐term and short‐term choice making and O'Donovan et al. (2017) with an Irish sample identified ‘everyday’ and ‘key life’ decisions. Service users with IDD have been shown to make more everyday choices than support‐related choices (Houseworth et al. 2018; Tichá et al. 2012). These findings may be due to support‐related choices being constrained by funding, regulations and agency‐level practices (e.g., staff hiring) and so be mostly beyond the influence of DSPs and service users, while everyday choices (e.g., daily routines) can be constrained or facilitated by the people directly involved.

As demonstrated by Friedman (2018), greater individual choice was facilitated by staff stability. In the current study, we only examined choice when the individual with IDD responded themselves and excluded proxy data. The rationale for this approach was to be as person‐centred as possible when measuring choice, community participation and staff turnover.

Community participation for many people with IDD is only possible with DSP support, such as support with transportation, exploring community activity options and participation in community activities. Friedman (2018) confirmed the importance of DSPs in supporting quality of life, including community participation of people with IDD. Similarly, Spassiani et al. (2019) identified the frontline workforce as a key factor in supporting community participation by people with IDD.

Stancliffe et al. (2023) reported that attending community groups and religious services were each strongly associated with better social participation outcomes among adults with IDD. However, 45.0% of their respondents indicated mainstream community‐group participation as an unmet need (Stancliffe et al. 2023). Staff turnover could explain why so many people report this unmet need. Experienced staff who know the person with IDD well and have developed a good working relationship with them are more likely to be aware of an individual's wishes regarding community participation, such as wanting to join a community group in an Australian context (Robinson et al. 2020; Topping et al. 2022). Frequent staff turnover likely undermines awareness and achievement of such goals. The measure of community participation used in the current study is person‐centred as it considers whether the frequency of a person's community participation accords with the individual's preference.

Both choice and community participation represent important outcomes that could be facilitated or inhibited by consistent and reliable DSPs. Further, person‐centred measures of both represent an opportunity to improve our understanding of how staff turnover directly and personally impacts service users with IDD. We recognize that DSP performance is one of many factors that could impact an individual's community participation but nonetheless can serve as an important facilitating factor for many individuals with IDD.

## 2 Research Questions and Purpose

The purpose of this study is to use the NCI‐IPS 2022–2023 data to explore (a) whether the individual's opportunity to choose their own staff is related to the perceived staff turnover they report, and (b) how an individual with IDD's perception of staff turnover is related to their community participation and choice. These analyses expand on previous work by Friedman (2018) on individual staff turnover and other work related to agency‐level staff turnover. We selected these two specific outcomes based on previous research (Friedman 2018; Houseworth et al. 2023; Spassiani et al. 2019) as a starting point in exploring the impact of staff turnover on outcomes experienced by people with IDD. We expand previous research by testing the inference that staff turnover is related to personal quality of life outcomes with person‐centred data.

This study addresses the following research questions:

1. Is individual choice of staff associated with perceived staff turnover?
2. Is perceived staff turnover associated with whether an individual's community participation matches their preferences?
3. Is perceived staff turnover associated with an individual's choice‐making opportunities?

## 3 Methods

### 3.1 Sample

The data we analysed came from the 2022–2023 NCI‐IPS, an outcomes measurement program that provides an annual national and state picture in the United States of the lives of adults with IDD service users in the US Participating states are asked to sample at random or in a stratified random fashion a minimum of 400 adults with IDD receiving publicly funded long‐term services and supports (NCI‐IPS 2023). Sampling strategies may vary by state (see Appendix B in the 2022–2023 IPS Final Report).

NCI‐IPS participants are 18 or older and receive at least one service from their US state IDD system in addition to case management. Participants in 2022–2023 came from 33 states (AL, AR, CA, CT, DE, DC, FL, GA, HI, IL, IN, KS, KY, MD, MI, MN, MO, MT, NE, NV, NH, NJ, NY, NC, OK, OR, PA, SC, TX, UT, VA, WI, WY). Recruited total samples averaged 770 per state and ranged from 77 (WY) to 5125 (CA). See the Weighting section below regarding the overrepresentation of some states, notably California, in our sample.

### 3.2 Analytic Sample

After listwise deletion across the dependent variable, independent variables, and covariates, and weighting, our final sample included 4301 cases for the person‐centred community participation. Table 1 below shows the characteristics for our final analytic sample. Other analyses included have slightly different sample sizes based on the variables included as listwise deletion was the method employed across analyses.

**TABLE 1:** Descriptive statistics for final analytic sample for person‐centred community participation.

Variable	Level	*N*	Percent
Level of ID	Moderate	1204	28.07%
	Severe	138	3.22%
	Profound	18	0.42%
	Mild*	2930	68.30%
Autism	Present	744	17.34%
	Not present*	3546	82.66%
Mobility	Mobile	3646	84.99%
	Non‐mobile*	644	15.01%
Verbal expression	Verbal	4139	96.48%
	Non‐verbal*	151	3.52%
Behavioural challenges	Present	2004	46.71%
	Not present*	2286	53.29%
Mental health diagnosis	Present	248	5.78%
	Not present*	4042	94.22%
Gender	Male	2401	55.97%
	Female*	1889	44.03%
Race/ethnicity	Other	210	4.90%
	Asian	115	2.68%
	African American	701	16.34%
	Latino	302	7.04%
	White*	2962	69.04%
Residential setting	Group home	1404	32.73%
	Own	953	22.21%
	Other	525	12.24%
	Family*	1408	32.82%
IV: Self‐perceived staff stability	Staff stable	2506	58.41%
	Staff not stable*	1784	41.59%
DV: Person‐centred community inclusion	Enough community inclusion	1442	33.61%
	Not enough community inclusion*	2598	60.56%

*Note:* *N* = 4290 for weighted final sample. Mean age = 41.41 (SD = 14.56). * = referent category.

Abbreviations: DV = dependent variable; IV = Independent variable.

Our decision to exclude individuals who could not reliably answer survey questions on their own almost certainly created a pattern of missing data that was not random. Basic comparisons reveal that excluded individuals had more severe levels of intellectual disability (ID) and were less able to express themselves verbally. Firstly, only 16,077 (63% of the full sample) were deemed capable of answering NCI‐IPS Section I items (all of which require self‐report) based on NCI‐IPS procedures. Those excluded were considered either cognitively unable to understand Section I questions or unable to respond reliably on their own. Next, 10,583 participants (66% of Section I respondents) indicated that they had staff present. Based on NCI‐IPS procedures, the remaining group are not queried about staff stability. Finally, 6537 (62% of those eligible to respond to the staff stability question) responded to the item. The remaining 38% were missing due to their staff being present in the room during the interview (a situation that requires the interviewer to skip the staff stability item), ‘don't know’ responses, or other undescribed ‘missing’ responses. Along with listwise deletion based on other items, this reduced our final analytic sample greatly from the full NCI‐IPS sample. However, this was a necessary trade off given the purpose of the study was to hear from the people with IDD receiving services.

### 3.3 Instrument

The NCI‐IPS was developed by the National Association of State Directors of Developmental Disabilities Services (NASDDDS) and the Human Services Research Institute (HSRI) (Bradley and Moseley 2007). The NCI‐IPS survey is used to collect cross‐sectional data on service users with IDD in the United States. Data analysed in this study came from all three sections of the NCI‐IPS. The Background Information Section includes questions related to personal demographics, disability diagnoses, health, mobility, communication, residence type and size, employment and behavioural supports. These items are typically completed by agency staff or case managers/service coordinators based on agency records but may also be completed by the individual or family members. Section I includes questions related to employment/daily activity, home, friends and family, safety, satisfaction with services and self‐direction. Section I is unique in that the items in it can only be answered by the individual receiving services. Proxy responses are not accepted to Section I items, which reduced our overall final analytic sample as our main independent variable, whether the individual felt their staff changed too often, came from this section. Section II includes items related to community participation, choices, rights and access to needed services. These items can be answered by the individual or a proxy. Individual responses were required to these items as well. Therefore, our sample was reduced from the total due to missing responses and items not or unable to be answered by the individual. Typically, around one third of NCI‐IPS participants have missing data for Section I items (Stancliffe et al. 2014).

### 3.4 Variables

#### 3.4.1 Dependent Variables

Three dependent variables reflecting community participation were included in our analysis. The person‐centred community participation scale was developed based on five person‐centred items in the Community Participation section of Section II of the NCI‐IPS. This section queries how often (e.g., once a week, twice a week, not at all) in the last month each participant engaged in each of the five types of community participation included in this scale: shopping, running errands, entertainment, community group activity, eating out. A follow‐up question was recently added (e.g., Think about how often you went shopping in the past month. Would you like to go shopping more, less, or the same amount as now?) to capture more person‐centred information. This follow‐up item essentially questions whether the frequency of community engagement matches the person's preferences. Response options included: ‘more’, ‘less’, or ‘the same amount as now’. For our scale, we coded the options ‘more’ or ‘less’ as 0 = not matched to the person's preference. Option ‘the same amount as now’ was coded as 1 = matched to the person. The rationale for this coding is as follows. A match between experienced and desired community participation is clearly positive. More frequent community participation may superficially seem better and less frequently worse, but only until one considers the person's activity preferences. For someone who is required to go shopping but finds it unpleasant, *more* frequent shopping would be even more unpleasant (i.e., more = worse), whereas less shopping would be preferred (less = better). Similarly, for a person who enjoys eating out, *less* of this activity would not be desired (i.e., less = worse) and more eating out would be positive (more = better). That is, the ‘less’ and ‘more’ options cannot be coded as unambiguously better or worse unless the individual's liking for the specific activity is known, data that is not collected by the NCI‐IPS. Therefore, we resolved this ambiguity by coding ‘less’ and ‘more’ equally as 0.

The areas of community participation in the scale were selected based on factor and reliability analyses. We conducted an exploratory factor analysis with equimax rotation and a cutoff of 0.5 One factor emerged from the factor analysis. Areas of person‐centred community participation included were shopping, entertainment, eating out, religious services and community group attendance. Cronbach's alpha was 0.69. Scores on each item (0 or 1) were averaged to create a scale between 0 and 1. A minimum of three items (60%) were required to be recorded for each participant to get a scaled score. The average score was 0.59 (SD = 0.33), range 0–1.

Given that most other variables in these analyses were categorical in nature, we dichotomized the Person‐centred community participation scale based on a median split. We decided on this dichotomization approach as research shows results often lead to the same conclusion as with continuous variables and given the categorical nature of the other variables included in this study, this decision aids in presenting the results in a consistent manner across all dependent and independent variables (DeCoster et al. 2009). Scores 0.6 or below = 0 community participation not matched and score above 0.6 = 1 community participation matched.

Support‐related and Everyday Choice scales were both developed based on previous work (Tichá et al. 2012; Houseworth et al. 2018) and factor analyses with loading cutoffs of 0.5 or above and reliability analyses. Scale scores were calculated by averaging individual item scores. For a scale score to be computed, at least 60% of the scale questions must have been answered. The range for both scales was 0–2. A mean score of 0 represented no choice, a score of 2 represented full choice and values in between represented some choice. Choice items came from Section II, which can be answered by the individuals or a proxy. To maintain our person‐centred focus, we excluded proxy‐based responses in our regression analyses of choice, meaning that all responses to choice items came from the person with IDD.

The support‐related choice scale included three items: (1) Who chose (or picked) the place where you live? (2) Did you choose (or pick) the people you live with (or did you choose to live by yourself)? and (3) Who chose (or picked) the place you work? and/or who chose (or picked) your day program or workshop? (Did you help make the choice?). These two items are not asked of people living with family, restricting the sample for this outcome. At least two items (67%) had to be completed for a scale to be computed. Cronbach's alpha was 0.77 for the support‐related choice scale. The average score was 1.04 (SD = 0.60). Note compared to previous work on NCI‐IPS choice scales, the ‘can you change your case manager’ item no longer reliably scaled for the support‐related choice scale and was therefore excluded. We also excluded the ‘do you choose (or pick) your staff?’ item as we were interested in exploring this separately as part of our analyses. This decision did not compromise the reliability of the scale.

The everyday choice scale included four items: (1) Who decides your daily schedule (like when to get up, when to eat, when to go to sleep)? (2) Who decides how you spend your free time? (3) Do you choose what you buy with your spending money? and (4) Who chose (or picked) the other regular activities you do? At least three items (75%) had to be completed for a scale to be computed. Cronbach's alpha was 0.75 for the everyday choice scale. The average score was 1.75 (SD = 0.34). Note, we added the new NCI‐IPS item ‘other regular activities in the community’ item as it factored well and maintained high reliability for the scale.

Given that most other variables in these analyses were categorical in nature, we dichotomized each choice scale based on a median split.

#### 3.4.2 Intermediate Variable (Independent and Dependent Variable)

- Self‐perceived staff stability was based on the question in Section I (Sometimes people's staff change, and new staff start working. Do your staff change too often? Do you get new staff too often?) with response categories (Yes, staff do change too often; Sometimes or some staff; No, staff do not change too often). ‘Yes’ and ‘sometimes’ responses were coded 0 = staff stability not perceived and ‘No’ responses were coded 1 = staff stability perceived. 60% of NCI‐IPS participants indicated staff stability while 40% indicated some staff instability.

#### 3.4.3 Independent Variables

- Choosing own staff was based on a single question ‘do you choose (or pick) your staff?’. Reponses were coded into three nominal levels: 1 = Someone else chose, 2 = staff are assigned but can be changed if requested by the person and 3 = person chose staff.

#### 3.4.4 Covariates

Based on previous research, we choose to adjust for the following variables from the Background Information Section:

- Age is a continuous item that provides the age of each participant at the end of the survey.
- Level of ID is a single item that categorizes ID into mild, moderate, severe and profound levels. Those categorized as unknown/other or with no ID diagnosis were excluded from analysis. Mild ID was the referent category.
- Autism indicates whether a participant has an autism diagnosis or not. No autism diagnosis was the referent category.
- Verbal expression was based on a question asking the participant's usual means of communication. The categories were coded into verbal communication or other. Non‐verbal was the referent category. Many of the group who do not communicate verbally were excluded due to NCI‐IPS Section I requiring self‐reports only, as described above in Section 3.2; however, a small group who communicate in other ways were included to factor out variance due to this difference.
- Mobility was based on the ability to move around freely without assistance. Individuals who need assistance to move around with aids or a wheelchair or who are non‐ambulatory and always need assistance served as the referent category.
- Behaviour Challenges was based on a series of three items assessing whether the participant needed support for various behaviours: self‐injurious, disruptive, or destructive. We collapsed these items into No behaviour needs and Any needs and added them together yielding a score that ranged from 0 to 3. No behavioural supports needed served as the referent category.
- Mental health diagnosis was based on three types of mental health conditions diagnosed: anxiety, psychosis, or other mental illness. The presence of any or all categories was coded yes, while the absence of all three was coded no. No mental health diagnosis served as the referent category.
- Gender was coded male (referent) and female. The small percentage defined as ‘other’ was not included in this analysis to explore gender.
- Race/ethnicity is a single item with multiple categories. We reduced these categories to White (referent), African American, Hispanic/Latino and other (i.e., Asian, Pacific‐Islander, and all remaining identifications).
- Residential Setting included multiple categories. We collapsed them into four categories, including people living in a group‐home setting of any size, people living with family (or foster or hosts), people living independently (own home/apartment) and people living in any other setting (including institutions, nursing homes, etc.). Family/host home served as the referent category. The information for this question comes from the Background Information section of the NCI‐IPS.

### 3.5 Weighting

Our final inferential model was weighted by state. NCI‐IPS 2022–2023 weights were calculated using each state's number of valid surveys and its total survey‐eligible population, so that each state's weighted contribution is proportional to its adult service population (National Core Indicators In‐Person Survey 2023). States randomly select service users to be surveyed. Most states sample from all adult service users, so a single state weight is applied to all participants from that state. Weighting was applied to all our analyses to adjust for the effect of oversampling in states.

### 3.6 Data Analysis

Descriptive statistics were run on all independent and dependent variables and covariates. Missing data was deleted listwise. Logistic regression models were run to explore the outcomes of person‐centred community participation, everyday choice and support‐related choice, as well as on a single item out of the support‐related choice scale related to being able to choose staff. Logistic regression was chosen since all dependent and independent variables were categorical (dichotomous). One model was run for each outcome. IBM SPSS version 29 was used to conduct the analyses (IBM Corp. 2022).

## 4 Results

### 4.1 Choosing Own Staff and Staff Stability

Table 2 below shows the results of the logistic regression model for staff stability, focusing on the role of choosing one's own staff as the primary independent variable.

**TABLE 2:** Logistic regression results for person‐centred staff stability (*N* = 4160).

	*B*	S.E.	Wald	df	Sig.	Odds ratio	95% CI for EXP(*B*)	
							Lower	Upper
Age (continuous)	0.01	0.00	4.57	1	0.03	1.001	1.00	1.01
Level of ID (mild referenced)			0.50	3	0.92			
Moderate	0.03	0.08	0.12	1	0.73	1.03	0.89	1.19
Severe	−0.04	0.20	0.03	1	0.86	0.96	0.65	1.43
Profound	−0.23	0.40	0.32	1	0.57	0.80	0.36	1.76
Autism present	−0.00	0.09	0.00	1	0.97	0.99	0.83	1.19
Mobility	0.36	0.10	13.32	1	< 0.01	1.43	1.18	1.74
Verbal expression	−0.48	0.18	7.27	1	0.01	0.62	0.44	0.88
Mental health present	−0.22	0.14	2.64	1	0.11	0.80	0.62	1.05
Behavioural challenges	−0.11	0.07	2.20	1	0.14	0.90	0.78	1.04
Gender male	0.04	0.07	0.37	1	0.54	1.04	0.91	1.19
Race (white referenced)			7.66	4	0.11			
Other	−0.14	0.13	1.05	1	0.31	0.87	0.67	1.13
Asian	0.01	0.25	0.00	1	0.97	1.01	0.62	1.64
African American	0.18	0.09	3.78	1	0.05	1.20	0.99	1.43
Latino	−0.18	0.14	1.70	1	0.19	0.84	0.64	1.09
Residential setting (family referenced)			36.92	3	< 0.01			
Group	−0.46	0.09	25.51	1	< 0.01	0.63	0.53	0.76
Own	−0.14	0.10	1.91	1	0.17	0.87	0.72	1.06
Other	−0.56	0.12	22.96	1	< 0.01	0.57	0.46	0.72
**Choose own staff (person chose referenced)**			**44.70**	**2**	**< 0.01**			
**Someone else chose**	**−0.62**	**0.09**	**42.83**	**1**	**< 0.01**	**0.54**	**0.45**	**0.65**
**Assigned but person can change**	**−0.49**	**0.09**	**29.08**	**1**	**< 0.01**	**0.61**	**0.51**	**0.73**
Constant	1.16	0.28	17.80	1	< 0.01	3.20		

*Note:* Effect size estimates 0.04 and 0.05 based on like Nagelkerke's and Cox and Snell's *R*‐squared. Bolded values indicate the variable is significant at *p* < 0.05.

The main independent variable in this model, the individual's self‐report of choosing their own staff, was a significant predictor of person‐centred staff stability. Individuals who did not choose their own staff were approximately 45% less likely to report that their staffing was stable.

### 4.2 Self‐Perceived Staff Stability

Overall, as shown in Table 1, 58% of NCI‐IPS participants indicated staff stability while 42% reported some staff instability. In all cases, individuals who indicated their staff did not change too much (staff stable) also indicated higher levels of everyday choice, support‐related choice and community participation (contact authors for more information), though these results varied when analysed via regression in the next set of analyses.

### 4.3 Person‐Centred Community Participation

Table 3 below shows the results of the logistic regression model for person‐centred community participation.

**TABLE 3:** Logistic regression results for person‐centred community participation (*N* = 4290).

	*B*	S.E.	Wald	df	Sig.	Odds ratio	95% CI for EXP(*B*)	
							Lower	Upper
Age (continuous)	0.00	0.00	0.70	1	0.40	1.00	0.99	1.01
Level of ID (mild referenced)			6.20	3	0.10			
Moderate	−0.17	0.08	5.11	1	0.02	0.84	0.72	0.98
Severe	−0.11	0.21	0.27	1	0.60	0.90	0.60	1.35
Profound	0.35	0.40	0.76	1	0.38	1.42	0.65	3.08
Autism present	−0.12	0.09	1.67	1	0.20	0.89	0.74	1.06
Mobility	0.08	0.10	0.59	1	0.44	1.08	0.89	1.32
Verbal expression	−0.41	0.16	6.32	1	0.01	0.67	0.49	0.91
Mental health present	0.19	0.14	1.82	1	0.18	1.21	0.92	1.60
Behavioural challenges	−0.33	0.07	20.81	1	< 0.01	0.72	0.62	0.83
Gender male	0.11	0.07	2.40	1	0.12	1.11	0.97	1.27
Race (white referenced)			5.97	4	0.20			
Other	−0.07	0.13	0.25	1	0.62	0.94	0.72	1.22
Asian	−0.02	0.25	0.01	1	0.93	0.98	0.60	1.60
African American	−0.21	0.09	4.95	1	0.03	0.81	0.68	0.98
Latino	−0.18	0.14	1.65	1	0.20	0.84	0.64	1.10
Residential setting (family referenced)			44.62	3	< 0.01			
Group	−0.24	0.09	6.59	1	0.01	0.79	0.66	0.95
Own	0.41	0.10	17.81	1	< 0.01	1.50	1.24	1.81
Other	0.01	0.12	0.01	1	0.93	1.01	0.80	1.27
**Staff stability**	**0.26**	**0.07**	**14.41**	**1**	**< 0.01**	**1.30**	**1.13**	**1.49**
Constant	−0.44	0.27	2.71	1	0.10	0.64		

*Note:* Effect size estimates 0.03 and 0.04 based on like Nagelkerke's and Cox and Snell's *R*‐squared. Bolded values indicate the variable is significant at *p* < 0.05.

Covariates with significant associations with person‐centred community participation include verbal expression, behavioural challenges and residential setting. Individuals verbally expressive and individuals with behavioural challenges were less likely to indicate their community participation matched their preferences. Regarding residential setting compared to those in family homes, individuals living in group homes were also less likely to report their community participation levels matched their preferences, while individuals living in their own homes were more likely to indicate matched community participation levels.

The main independent variable in this model, the individual's self‐report that staff change too much, was a significant predictor of person‐centred community participation. Individuals who indicated their staff were stable were 30% more likely to indicate their community participation levels matched their preferences.

### 4.4 Self‐Reported Choice Making Opportunities

For the support‐related choice scale, the average score was 1.04 (SD = 0.60) and for the everyday choice scale, the average score was 1.75 (SD = 0.34). Neither model for choice‐making opportunities was significant for staff stability. To conserve space, we do not present these models in this manuscript but they can be provided upon request.

## 5 Discussion

### 5.1 Choice of Staff and Self‐Perceived Staff Stability

This study contributes important information to the gap in literature on the choice of staff by adults with IDD and perceived staff turnover. A limited number of qualitative studies have shed some light on the importance of selecting one's own staff on satisfaction with their services, including with staff themselves (Pallisera et al. 2021; Swaine et al. 2016). We found a significant positive relationship between individual service users' level of choice related to their staff selection and their self‐reported staff stability. As far as we know, this is the first quantitative analysis to examine the relationship between choice of staff and perceived staff stability. These results indicate that having a role in choosing your staff is related to (although not strongly) staff stability from the person's perspective. It is likely that individuals who have a role in the hiring or placement process are likely to select staff that they feel comfortable with and who meet their individual needs better, factors that presumably promote both staff stability (Murray et al. 2022) and individual outcomes.

One group of service users who can select staff are those who use self‐directed funding (SDF). One key element of SDF is ‘employer authority’ which involves the freedom to select or hire, train, supervise, schedule and in some cases to dismiss one's support professionals (e.g., Friedman 2025). Further, employer authority often includes the option of hiring family members or friends. It is likely that having family or friends as paid staff would enhance staff stability because of their pre‐existing relationship with the service user (Robinson et al. 2020; Topping et al. 2022).

### 5.2 Self‐Perceived Staff Stability

Overall, 58% of NCI‐IPS participants in the current study indicated they perceive their staff as stable while 42% indicated some perceived instability. This result differs somewhat from a similar study, using a different, objective data source, where 56% of respondents with IDD had experienced a change in direct staff in the last 2 years (Friedman 2018). A possible reason for our seemingly lower rate of instability is that perceived staff stability is likely to reflect the importance of the specific staff change to the individual. If the person's relationship with a DSP is superficial and functional, then the replacement of that DSP with another may have little impact. There is an important distinction between Friedman's (2018) objective data about an individual's exposure to staff turnover and the current study, as our results are based solely on the self‐reported perception of the individual receiving services regarding whether they feel that staff change too often.

Our results indicate that from the perspective of many service users with IDD, there is a problem with staff turnover. These results are particularly important because they are not just based on objective staffing change as indicated by a calculated staff turnover, but whether the person feels it is sometimes or definitely happening ‘too often’, indicating personal discontent. The reason the individual's subjective perspective is important is because it captures the subjective impact of staff change on the individual. For example, if the individual has a deep, trusting relationship and confides in a particular DSP, when that DSP leaves this staff change may have a considerable impact.

### 5.3 Self‐Perceived Staff Stability and Community Participation

We found that subjective staff stability was related to more person‐centred community participation. Individuals who reported staff changed too often also were more likely to indicate their level of community participation did not match their desired levels. These results complement similar findings by Friedman (2018) who based her results on objective staff stability. Our research suggests that subjective staff stability has positive effects on community participation, impacting the rights of individuals with IDD to interact with the world around them as often as they would prefer. When subjective staff stability is present, individuals with IDD are more likely to achieve positive person‐centred community outcomes, in this case individually preferred levels of access to the community for various common activities, such as shopping, entertainment, eating out, religious services and community group attendance. Staff turnover may be related to new staff not knowing the people with IDD they serve well and thereby being less informed and skilled in supporting individual preferences. We do note that we detected a modest effect size for this finding, indicating staff stability is only one of several factors related to perceived community participation by individuals with IDD.

### 5.4 Self‐Perceived Staff Stability and Choice‐Making

We hypothesized that choice‐making would be another outcome impacted by perceived staff stability. Unlike Friedman (2018), our analyses, however, did not detect a relationship between staff stability and the two types of choice‐making assessed, every day and support‐related choice. One reason for this finding may be due to a ceiling effect in some choice data we analysed. For everyday choice making, even before restricting the sample to individuals who can answer for themselves (i.e., those with milder levels of ID), average scores already approach 2, the maximum score on that scale. As such, a ceiling effect leaves little variability for a factor such as staff stability to significantly explain, especially when we examined a sample that had milder disability levels and may have lived in more favourable settings for everyday choice making compared to the whole sample. However, a ceiling effect was not evident for support‐related choice, so it cannot account for the non‐significant finding in that case.

In terms of explaining this choice result compared to community participation, community participation may depend upon a more reliable presence of a staff person than choice. For many individuals with IDD, many aspects of choice, especially those with fewer support needs, should be autonomous. This is especially true for everyday choices, such as choosing how to spend free time. Community participation, however, often requires more direct supports, such as transportation, for most individuals with IDD.

## 6 Limitations

One limitation of this study is that the cross‐sectional nature of this analysis did not allow for causal conclusions. Next, data are limited to service users with IDD who can reliably respond for themselves, a subset who typically have milder ID, communicate using speech and do not need extensive support for behaviour (Stancliffe et al. 2014). Results may not generalize to the entire population, including individuals with the highest support needs and are not relevant to people with IDD not receiving services.

Two items on the support‐related choice scale (Who chose the place where you live?; Did you choose the people you live with?) are not asked of people who live with family. Therefore, this scale was primarily defined by those not living with family.

Other important factors likely impact an individual's community participation (e.g., social capital, familial support). However, the survey used in the current study is limited in its scope, not allowing for exploration of these important other factors.

Finally, our methods reduced our sample substantially from the full NCI‐IPS sample, skewing our results to people with milder levels of ID. Our results may not generalize to the larger population of individuals with IDD receiving services.

## 7 Future Directions

Given that models of funding for services for people with IDD are changing, including self‐directed services, increasing autonomy in selecting services and staff who provide those services, these issues are important areas for future research. It will also be important to understand the relationship between staff turnover as perceived by the individual with IDD and objective measures of staff turnover. Additional research is needed on choosing staff as it relates to staff turnover and in turn to making choices in daily living, longer‐term decisions, as well as to additional outcomes not examined in this study (e.g., transportation, employment and relationships). Future research should include longitudinal analyses that explore causal relationships between improvements in staff stability and quality‐of‐life outcomes.

Next, future research should consider other factors related to community participation such as social capital to place results into a broader context.

Further, future research should consider systematically exploring how choosing staff relates to quality‐of‐life outcomes. We found a relationship between choosing staff and turnover. But there remains work to be done to better explore paths to quality‐of‐life outcomes that begin with an individual service user having a role in choosing the staff that assist them regularly.

Finally, research should evaluate whether the relationship between choice of staff and stability of relationships may be stronger when the staff person chosen is someone that they already know and have a relationship with, such as friends or family. These data were not available for the current study.

## 8 Conclusion

The current study is unique in that it relies on the voices of people with IDD receiving services. We found that when individuals are involved in the hiring and/or placement process of staffing, they are more likely to report better staff stability. Previous research has assumed staff turnover affects many quality‐of‐life outcomes. Our results suggest from an individual perspective, this relationship depends on the specific outcome being assessed. We found that a person's perception of staff stability is related to whether a person's desired level of community participation is met. These results can inform more recent models of service provision in which the person with IDD is directing their own services, including hiring of staff, in the US and internationally. Finally, we found a significant relationship between individual services users' level of choice related to staff and their self‐reported staff stability. This is an important newly identified factor that contributes to staff stability.

## Funding

This work was supported by National Institute on Disability, Independent Living, and Rehabilitation Research, 90DNCE0007‐01‐00.

## Ethics Statement

The University of Minnesota's institutional review board (IRB) reviewed this research and granted a waiver of ongoing IRB review and approval.

## Conflicts of Interest

The authors declare no conflicts of interest.

## Data Availability

The data that support the findings of this study are available from HSRI. Restrictions apply to the availability of these data, which were used under license for this study. Data are available from https://www.hsri.org/ with the permission of HSRI.
